# Update on intestinal ultrasound in pediatric inflammatory bowel disease

**DOI:** 10.1007/s00431-026-07194-w

**Published:** 2026-06-27

**Authors:** Gábor A. Dunay, Jan Däbritz

**Affiliations:** 1 Department of Pediatrics, Pediatric Gastroenterology, University Medical Center of the Brandenburg Medical School (MHB), Brandenburg an der Havel, Germany; 2 Faculty of Health Sciences, Joint Faculty of the Brandenburg University of Technology Cottbus-Senftenberg, Brandenburg Medical School Theodor Fontane and University of Potsdam, Potsdam, Germany; 3 Department of Pediatrics, Greifswald University Medical Center, Ferdinand-Sauerbruch-Str. 1, Greifswald, Germany; 4 Department of Pediatrics, Klinikum Ernst Von Bergmann, Potsdam, Germany; 5 Institute for Clinical Research and Systems Medicine, HMU Health and Medical University, Potsdam, Germany

**Keywords:** Crohn’s disease, Ulcerative colitis, Intestinal ultrasound, Bowel wall thickness, Mesenteric fat, Children

## Abstract

**Purpose:** Intestinal ultrasound (IUS) is an accessible imaging tool in pediatric inflammatory bowel disease (IBD), its validation and standardization are a topic of intensive ongoing research. Here we provide an update on recent evidence including interim results from new studies. **Methods: **Literature search was performed using PubMed and OpenEvidence on IUS in pediatric IBD and the following subtopics: (1) validation of IUS in pediatric IBD; (2) bowel wall thickness (BWT); (3) Doppler-based evaluation; (4) mesenteric fat; (5) fibrostenosis and intestinal strictures; (6) comparison of different scoring systems; and (7) IUS in pediatric IBD monitoring. Furthermore, conference abstracts and poster presentations from major conferences were screened to identify ongoing studies with relevant preliminary findings. **Results: **IUS specificity and sensitivity for inflammation was found to be above 80–90% for the bowel segments accessible to ultrasound (colon, terminal ileum), but more limited for small bowel. Consensus is emerging for a threshold value of < 2.5 mm for BWT in deep remission of pediatric IBD, which is lower than in adults. In multiple studies, BWT alone correlated as well as more complex IUS-based scores with endoscopy and laboratory markers of inflammation. The Chicago Mesenteric Fat Index (CMFI) was developed for quantification of mesenteric fat and may improve standardization and reproducibility in research and clinical applications.

*Conclusion*: Significant recent progress in standardization and validation of IUS in pediatric IBD will most likely lead to its widespread application in outpatient and bedside monitoring including possible adoption of IUS based endpoints in clinical studies.

**Table:** 

**What is Known:** •* Intestinal ultrasound is gaining importance in the diagnostics and management of pediatric inflammatory bowel disease.*
**What is New:** • *Simplified ultrasound assessment measuring bowel wall thickness alone may be as informative as more complex scores. A consensus new cut-off value < 2.5 mm for bowel wall thickness in children is emerging.* • *A standardized quantification system called the Chicago Mesenteric Fat Index in pediatric inflammatory bowel disease has been proposed.*

**What is Known:**

•* Intestinal ultrasound is gaining importance in the diagnostics and management of pediatric inflammatory bowel disease.*

**What is New:**

• *Simplified ultrasound assessment measuring bowel wall thickness alone may be as informative as more complex scores. A consensus new cut-off value <  2.5 mm for bowel wall thickness in children is emerging.*

• *A standardized quantification system called the Chicago Mesenteric Fat Index in pediatric inflammatory bowel disease has been proposed.*

## Introduction

Intestinal ultrasound (IUS) in the imaging of inflammatory bowel disease (IBD) has emerged as a non-invasive and inexpensive tool in diagnosis and monitoring, especially in children. Cross-sectional imaging in IBD includes magnetic resonance enterography (MRE) as well as IUS. These modalities have an essential role in the evaluation of small bowel disease and transmural healing, which associated with better long-term patient outcomes than mucosal healing as determined by endoscopy [1]. Several recent review articles have emphasized the advantages of IUS in the diagnosis and monitoring of IBD in adults [2] and children [3, 4]. IUS correlated well with endoscopic and biochemical markers of disease activity in all age groups. However, a lack of standardization [5] is a generally recognized limitation. For children, a standardized IUS-based treatment monitoring algorithm has already been proposed in 2023 by expert consensus [4]. A meta-analysis performed in 2022 compared MRE with IUS in a pediatric population found MRE to be superior based on pooled sensitivity and specificity [6]. Still, MRE has several disadvantages in the pediatric population such as large amounts of contrast fluid to be ingested and the need for children to remain still for an extended period, both limiting its use in the very young. Importantly, per patient costs of IUS are markedly lower than those of MRE [7].

Meanwhile, several very recently published or ongoing studies are addressing validation of IUS as compared to endoscopy, clinical scores and MRE (Fig. 1). The aim of this narrative review article is to summarize recent evidence on the dynamically evolving field of IUS in pediatric IBD, including abstracts presented at major conferences in 2025 and 2026.

## Methods

Based on two recent reviews [3, 4], the following subtopics of interest regarding IUS in pediatric IBD were identified: (1) validation of IUS in the diagnosis and monitoring of pediatric IBD through comparisons to other imaging modalities, endoscopy, and clinical scores; (2) cut-off for bowel wall thickness (BWT); (3) Doppler-based evaluation and scores; (4) mesenteric or creeping fat; (5) fibrostenosis and intestinal strictures; (6) comparison of different scoring systems; and (7) application of IUS in pediatric IBD monitoring. A search of the PubMed database was performed using relevant keywords of the above-mentioned subtopics: “pediatric IBD", “ultrasound", “children", “IUS", “validation", “bowel wall thicknes"s, “doppler signal", “mesenteric fat", “creeping fat", “fibrostenosis", “intestinal strictures", “scoring systems", “disease monitoring". Time filter was set to include publications since 2023, studies already reviewed in [3, 4] were excluded. Furthermore, the OpenEvidence AI tool was queried to present recent literature regarding the use of IUS in pediatric IBD relevant to each of these topics. Abstracts from major congress meetings pertaining to the topic of IUS in pediatric IBD were identified for inclusion in this review article: the annual congresses of the European Crohn’s and Colitis Organisation (ECCO) 2025 and 2026; Digestive Disease Week (DDW) 2025; European Society of Paediatric Gastroenterology, Hepatology and Nutrition (ESPGHAN) 2025; United European Gastroenterology Week (UEGW) 2025 as well as the International ESPGHAN-Symposium on Pediatric Inflammatory Bowel Disease (PIBD) 2025.

## Results

### Validation of IUS for diagnosis and monitoring of pediatric IBD through comparisons to other imaging modalities, endoscopy and clinical scores

When compared to endoscopy, IUS showed a sensitivity of 76% and specificity of 84%, which were better for moderate-to-severe disease, and good correlations to fecal calprotectin (FCP) [8]. In a cohort of 177 patients with a mean age of 13.3 years, the authors found these metrics to be even better, with a sensitivity of 92% and a specificity of 96% for IUS when compared to endoscopy [9]. In a recent study of 72 pediatric patients (CD, UC and IBD-U), IUS had a sensitivity of 88% and a specificity of 67% when compared to endoscopy in CD, while in UC the metrics were even higher with 91% and 100% respectively [10]. In comparison, the sensitivity and specificity of the clinical score Pediatric Crohn’s Disease Activity Index (PCDAI) to predict mucosal healing es measured by endoscopy were 63% and 77%, respectively, and for the weighted PCDAI (wPCDAI) 58% and 84% [11]. Correlations of IUS to clinical disease activity indices (Pediatric Ulcerative Colitis Activity Index [PUCAI] and PCDAI) appear to be weaker [8]. However, in the larger cohort mentioned above [9], these were also significant for PCDAI vs. International Bowel Ultrasound Segmental Activity Score (IBUS-SAS) for Crohn’s disease (CD) and PUCAI vs. Milan Ultrasound Criteria (MUC) for ulcerative colitis (UC). Of note, these studies used different IUS-based scoring systems and combinations of BWT, hyperemia, surrounding fatty proliferation and loss of wall stratification [8, 9].

Ongoing studies presented this year at major gastroenterology-related conferences highlighted differences in the diagnostic accuracy of IUS as compared to MRE in CD depending on the anatomic location. While findings of IUS and MRE seem broadly concordant [12–14], IUS may be more accurate in detecting inflammation and complications in the colon [15, 16] and terminal ileum (TI) [15–17] than in the proximal small intestine when compared to MRE. In a study done in 20 pediatric CD patients undergoing ileocecal resection, IUS findings were in complete agreement with intraoperative findings in 90% of the cases and only lesions proximal to the TI were missed, underlining the somewhat limited accuracy of IUS for the more proximal small intestine [18]. A recent study in 29 children with CD found that even for TI, sensitivity of IUS may differ according to different segments, even though positive predictive value of IUS was high when compared to endoscopy [19]. Two independent studies have shown a good correlation between IUS and MRE when assessing BWT [15, 20]. In a recent retrospective analysis of 47 children with CD, ultrasound within 60 days of diagnosis detected small-bowel lesions in 15% of the children that were not otherwise seen on other modalities (MRE, endoscopy, histology), but it also missed lesions in 30% of the cases [21].

The diagnostic yield of IUS may be increased by contrast ultrasonography (small intestinal contrast ultrasonography [SICUS]), possibly providing accuracy for small bowel lesions that is comparable to MRE in pediatric small bowel CD [22]. Furthermore, combination of imaging modalities (IUS and MRE) can increase sensitivity and specificity when compared to reference standards such as endoscopy and histopathology [23].

### Bowel wall thickness

An increase in BWT is a hallmark of active IBD and is a parameter that is incorporated in all published IUS-based scoring systems [24–29]. Recent data regarding BWT cut-off values for pediatric IBD cohorts is summarized in Table 1 and discussed below.

**Table 1:** Bowel wall thickness in pediatric inflammatory bowel disease

**Bowel segment** (reference standard)	**BWT** Cut-off/median [mm]	**IBD type**	**Specificity**	**Sensitivity**	**References**
Ileum and colon (endoscopy)	> 1.9	CD and UC	76%	64%	Chavannes et al. 2024 [31]
Colon (endoscopy)	≥ 2.5 (moderate/severe)	UC	94%	66%	Hudson et al. 2024 [30]
Colon (endoscopy)	≥ 3.5 (severe)	UC	86%	92%	Hudson et al. 2024 [30]
All bowel segments (endoscopy and MRE)	< 2.5 (inactive)	CD and UC	n/a	n/a	Kellar et al. 2025 [32]
Ileum and colon	= 2.0 = 1.0–1.6 (healthy control)	CD and UC	n/a	n/a	Khan et al. 2025 [8]
Mixed bowel segments	AI analysis: = 3.2 (active) = 1.2 (inactive) Manual analysis: = 3.1 (active) = 1.3 (inactive)	CD and UC	n/a	n/a	Kumaralingam et al. 2025 [33]
Terminal ileum	> 2.4	CD	84%	87%	Vos et al. 2025 [17, 37]
Proximal ileum (MRE)	> 2.0	CD	93%	87%	Vos et al. 2025 [17, 37]
Ileum	> 2.4	CD	n/a	n/a	Patel et al. 2025 [38]
Colon	> 2.3	CD	n/a	n/a	Patel et al. 2025 [38]
Colon (FCP > 250 µg/g)	> 2.5	UC	n/a	n/a	Patel et al. 2025 [38]
Terminal ileum and colon	≥ 3.0 (boys) ≥ 2.4 (girls)	CD and UC	85% 90%	61% 73%	Räisänen et al. 2026 [40]
Colon (endoscopy)	≥ 2.2 (< 6 years)	VEO-IBD	87%	55%	Räisänen et al. 2026 [40]
Colon and ileum (endoscopy)	> 2.0 (≤ 12 years)	IBD and non-IBD	93%	85%	Kullmann et al. 2026 [41]

*BWT*, bowel wall thickness; *IBD*, inflammatory bowel disease; *CD*, Crohn’s disease; *UC*, ulcerative colitis; *AI*, artificial intelligence; *MRE*, magnetic resonance enterography; *FCP*, fecal calprotectin; *VEO*, very early onset; *n/a*, data not available/reported

A recent work suggested that measurements of BWT alone may be as useful as composite ultrasound scores to assess disease severity in pediatric IBD [30]. Generally, a BWT of < 3 mm is accepted to differentiate normal from pathological bowel, but evidence emerging in recent years already suggested that in the pediatric population, normal BWT may be lower. Cut-off values for children for diagnosis and monitoring of therapy response, however, have not thus far been defined [3, 4].

A recent study in 33 children suspected of having IBD looked at the correlation between BWT as measured by IUS compared to the Simple Endoscopic Score for Crohn’s Disease (SES-CD) and the Ulcerative Colitis Endoscopic Index of Severity (UCEIS) for UC [31]. The authors computed an optimal BWT cut-off of 1.9 mm in this cohort, with a sensitivity of 64% and a specificity of 76% as compared to endoscopy. In another study enrolling 52 pediatric patients with suspected UC, a BWT cut-off of  ≥ 2.5 mm for moderate/severe endoscopic inflammation (sensitivity 66%, specificity 94%) and ≥ 3.5 mm for severe endoscopic inflammation (sensitivity 92%, specificity 86%) has been suggested [30]. A larger multi-center retrospective study involving 98 children specifically aimed at defining normal BWT in children with IBD in sustained deep endoscopic remission/transmural healing found that 99% of analyzed bowel segments were < 2.5 mm in thickness, noting that this is lower than the threshold of 3 mm in adults [32]. In another cohort of 50 children (25 with IBD and 25 healthy controls) median BWT values of 2 mm for ileum and colon in IBD patients and 1–1.6 mm for the control group [8] were determined. An interesting retrospective study employed machine learning on a dataset of 260 pediatric patients with IBD, with 1478 IUS images previously labeled abnormal and 3087 labeled normal [33]. The artificial intelligence (AI) was trained on a subset of annotated IUS images to recognize lumen/mucosa and the muscularis/serosa interfaces and then determined average BWT. The authors determined a median BWT of 3.05 mm for abnormal and 1.30 mm for normal test images when measured manually, whereas the automated analysis reported 3.18 mm and 1.19 mm, respectively.

Otani et al. introduced the sum of adjusted BWT (saBWT) to increase specificity and sensitivity of IUS as compared to endoscopy in a study with 40 children with UC [34]. This saBWT is a mathematically corrected sum of the representative BWT of all colonic segments (ascending colon, transverse colon, descending colon, sigmoid colon). Using this method, the authors achieved a similar sensitivity but a higher specificity when compared to endoscopy than by using maximum BWT or sum of BWT. Notably, both sensitivity and specificity of saBWT was higher than that of FCP (cut-off 773 μg/g) compared to endoscopy.

Hong et al. evaluated the use of the submucosal thickness (SMT) to BWT ratio (SMT/BWT) previously established in adults with CU [35] in a cohort of 132 IBD patients < 18 years (majority CD) [36]. The authors found that an SMT/BWT ratio of ≥ 0.5 correlated with endoscopic inflammation in UC, noting that this cut-off aligns with adult studies. For CD, however, no significant correlation between SMT/BWT and endoscopic activity was found.

A study involving 78 pediatric CD patients compared IUS to MRE and identified a BWT cut-off of 2.44 mm for the TI (sensitivity 87%, specificity 84%) and 2.02 mm for the proximal ileum (sensitivity 87%, specificity 93%) for active disease [17, 37]. Using MRE as a standard of comparison, the authors of a further recent study with 37 pediatric patients (both CD and UC) found the agreement between MRE and IUS to be 70% using an IUS BWT cut-off of 2 mm, 72% using 2.5 mm, and 74% using 3 mm [16]. Another study included 92 pediatric patients, both CD and UC, and identified a BWT best predicting a FCP > 250 µg/g. An ileal BWT cut-off was determined at > 2.4 mm for ileal or ileocolonic CD and a colonic cut-off of > 2.3 mm for ileocolonic or colonic CD. For UC patients, the BWT threshold was 2.5 mm to predict a FCP > 250 µg/g [38]. In a study with 40 pediatric UC patients, BWT alone correlated well with PUCAI as well as FCP and was able to distinguish active from inactive disease at a BWT cut-off of 3 mm (sensitivity 97%, specificity 71%), even though the authors did not see additional diagnostic value compared with FCP [39].

Importantly, normal values for BWT may differ across age-groups and sexes. One study involving 133 children (UC, CD as well as very-early-onset IBD) determined the BWT in male and female patients separately [40]. With a total of 147 IUS examinations with matched colonoscopies, the authors proposed in their paper detailed BWT cut-off values based on age, sex, type of disease, and anatomic location. The authors reported considerably lower sensitivity and specificity to detect inflammation, even with lower cut-offs, for the VEO-IBD disease group (< 6 years). In a study of 35 patients (30 IBD, 5 non-IBD) ≤ 12 years, a BWT cut-off of < 2 mm was found to be optimal, with the 2.5 mm cut-off producing a false negative rate of 62% [41].

### Doppler-based evaluation and scores

Evaluation of bowel-wall hyperemia using color-doppler measurements in IUS is a well-established parameter of inflammation and included in most IUS scoring systems [3, 4]. The system proposed decades ago by Limberg [42] is still widely used as a semi-quantitative measurement of the penetration of the Doppler-signal into the submucosa or the muscularis propria. A recently published study of 25 CD patients under 21 years evaluated the performance of a novel color-doppler based determination of bowel-wall and mesenteric vessel density in correlation with the (modified) Limberg-score and BWT, contrast-enhanced ultrasound as well as clinical inflammatory markers [43]. Only BWT and none of the Doppler-based measurements showed significant correlation with systemic markers of inflammation (erythrocyte sedimentation rate, C-reactive protein). The larger study by Patel et al. also concluded that adding Doppler-based measurements vs. BWT alone did not further enhance the predictive accuracy of IUS when correlated to clinical scores and FCP [38].

### Mesenteric or creeping fat

While the presence of increased quantities of mesenteric fat (also termed creeping fat) has long been recognized as a feature of active IBD, reliable or reproducible quantification techniques for IUS are missing so far [3, 4]. A recently published study on a small group of adult CD patients concluded that at least a binary determination (presence/absence) of mesenteric fat showed good inter- and intra-operator reproducibility [44]. For inclusion in larger studies, however, standardized measurements or a quantitative scoring system would be highly desirable.

Recognizing this need, the Chicago Mesenteric Fat Index (CMFI) has been recently proposed. The score defines three quantitative stages of mesenteric fat wrapping, these being < 25% of bowel-wall circumference (none 0), incomplete circumferential with skipped areas in the TI (incomplete 1) and complete circumferential with continuous presence along the TI (complete 2) [45, 46]. The system was tested in 48 IUS scans of 36 pediatric CD and was shown to be well reproducible. The score showed a good correlation to increased BWT (> 3 mm), mean BWT, loss of bowel-wall stratification and hyperemia. Correlations with clinical or endoscopic scores were not reported.

### Fibrostenosis and intestinal strictures

Fibrostenosis, especially of the TI, is a potentially irreversible complication of pediatric IBD, especially in CD. According to previous reports, IUS could not reliably differentiate fibrostenosis from inflammatory narrowing of the bowel lumen in IBD [3, 47, 48]. A recent review article showed a sensitivity of 70%, specificity of 98%, and overall accuracy of 88% for IUS in detecting small bowel stenosis in CD when compared to double balloon endoscopy [49]. Interestingly, detection of stenosis by ultrasound was more likely to be successful in younger patients and ileocolonic localization, which could be relevant to the pediatric population; however, studies performed in pediatric cohorts are not available.

### Performance of different scoring systems

To date, several different scoring systems exist for the evaluation of disease activity by IUS in children and adults with IBD. The scores developed for use in the pediatric population are Pediatric Crohn Disease Intestinal Ultrasound Score (PCD-US) [24] for CD; the UC Ultrasound Score/Civitelli Index [25] as well as the Ulcerative Colitis Intestinal Ultrasound Index (UC-IUS) [26, 27] for UC and the Simple Pediatric Activity Ultrasound Score (SPAUSS) [28] which may be used in both pediatric CD and UC. The available scoring systems for pediatric and adult IBD patients are summarized in Table 2, which includes a detailed description of each score. All of the above scores except for PCD-US have been externally validated in at least one cohort.

**Table 2:** Intestinal ultrasound-based score systems in pediatric inflammatory bowel disease

**Score** [Reference]	**IBD type**	**Age group**	**Formula**	**Variables**					
SUS-CD [57]	CD	Adults	Sum of BWT [mm] + Limberg score for all segments	BWT measured at thickest point Limberg-Score: 0–4 Segments: Rectum, left colon, transverse colon, right colon, TI					
IBUS-SAS [51]	CD	Adults	4 X BWT [mm] + 15 X intestinal fat + 7 X CDS + 4 X BWS	BWT: graduation mean of four two-dimensional measurements					
				Score	0	1	2	3	
				Mesenteric fat	Absent	Uncertain	Present		
				CDS	Absent	Short signals	Long signals inside bowel	Long signals inside and outside bowel	
				BWS (loss of)	Normal	Uncertain	Focal </= 3 cm	Extensive > 3 cm	
PCD-US [24]	CD	Children	Sum of points for each bowel segment	TI		Colon			
				BWT 2.0–3.0 mm: 1 point		BWT 1.6–2.0 mm: 1 point			
				BWT 3.0–3.7 mm: 2 points		BWT 2.0–2.7 mm: 2 points			
				BWT > 3.7 mm: 3 points		BWT > 2.7 mm: 3 points			
						Mesenteric fat infiltration: 1 point			
MUC [29]	UC	Adults	1.4 × BWT [mm] + 2 × CDS	BWT: average of 3 measurements CDS: 0 absent, 1 present					
UC-US/Civitelli-Score [25]	UC	Children	Sum of points for each bowel segment	1 point each of BWT > 3.0 mm, CDS present, loss of BWS, loss of Haustra Segments: left colon, transverse colon, right colon					
UC-IUS [26, 27]	UC	Children	Sum of points for each bowel segment	Score	1		2	3	
				BWT [mm]	> 2		> 3	> 4	
				CDS	Spots		Stretches		
				Haustrations	Abnormal				
				Mesenteric fat	Present				
				Colonic segments: rectum, sigmoid, descending colon, transverse colon, ascending colon					
SPAUSS [28]	CD and UC	Children	Sum of points for each bowel segment	Score	0	1	2	4	6
				BWT [mm]		1–3.9		4.0–6.9	≥ 7.0
				CDS	Absent	Mild	Moderate/severe		
				Mesenteric fat	Absent	Mild			Moderate/severe
				Segments: rectum, sigmoid colon, descending colon, transverse colon, right colon, TI					

*IBD*, inflammatory bowel disease; *CD*, Crohn’s disease; *UC*, ulcerative colitis; *SUS-CD*, simple ultrasound score for Crohn’s disease; *IBUS-SAS*, international bowel ultrasound segmental activity score; *MUC*, Milan ultrasound criteria; *PCD-US*, pediatric Crohn’s disease intestinal ultrasound score; *UC-US*, ulcerative colitis ultrasound score; *UC-IUS*, ulcerative colitis intestinal ultrasound score; *SPAUSS*, simple pediatric activity ultrasound score; *BWT*, bowel wall thickness; *BWS*, bowel wall stratification, *CDS*, color doppler signal; *TI*, terminal ileum

Recent studies on the performance of various IUS-based scores in pediatric IBD cohorts are summarized in Table 3. A recent publication by Hudson et al. compared UC-IUS, SPAUSS and Civitelli index, as well as the MUC [29] and BWT alone in a cohort of pediatric patients with UC [30]. From the scores tested, UC-IUS and the MUC demonstrated the strongest correlation with endoscopic disease activity. A more recent study in 75 pediatric UC patients similarly compared different IUS-based scores (MUC, Civitelli, and UC-IUS) to endoscopic disease activity. The authors found IC-IUS to be highly specific but less sensitive (0.9 and 0.54, respectively), while the Civitelli score was more sensitive but less specific (0.82 and 0.6) [50]. In a recent study including 78 pediatric CD patients the performance of BWT alone, IBUS-SAS, PCD-US, and SPAUSS were tested against MRE as a standard of comparison. From the scores tested, PCD-US had the overall best performance but did not outperform BWT alone [37].

**Table 3:** Overview of recent studies addressing the performance of IUS scores in pediatric IBD cohorts

Study [reference]	Population (*n*)	Scores compared	Reference standard	Key findings
Hudson et al. [30]	UC (52)	UC-IUS, SPAUSS, Civitelli, MUC, BWT alone	Endoscopic activity	UC-IUS and MUC strongest correlation with endoscopic disease activity
Massironi et al. [50]	UC (75)	UC-IUS, Civitelli, MUC	Endoscopic activity	UC-IUS: specificity 90%, sensitivity 54%; Civitelli: specificity 60%, sensitivity 82%
Vos et al. [37]	CD (78)	BWT alone, IBUS-SAS, PCD-US, SPAUSS	MRE	PCD-US best performance but did not outperform BWT alone
Benedetto et al. [52]	UC (77)	IBUS-SAS (adult scoring system)	Endoscopic activity	Good correlation between IBUS-SAS and endoscopic activity for moderate inflammation; IBUS-SAS > 48.5 showed significantly lower colectomy-free survival

*UC*, ulcerative colitis; *CD*, Crohn’s disease; *UC-IUS*, ulcerative colitis intestinal ultrasound score; *SPAUSS*, simple pediatric activity ultrasound score; *MUC*, Milan ultrasound criteria; *BWT*, bowel wall thickness; *IBUS-SAS*, international bowel ultrasound segmental activity score; *PCD-US*, pediatric Crohn’s disease intestinal ultrasound score; *MRE*, magnetic resonance enterography

An ongoing study evaluated the performance of the adult UC scoring system IBUS-SAS [51] in pediatric UC patients, analyzing 77 IUS studies and found a good correlation between IBUS-SAS and endoscopic activity for moderate inflammation, while an IBUS-SAS score > 48.5 showed a significantly lower colectomy-free survival [52].

### Application of IUS in pediatric IBD monitoring

Based on accumulating evidence on the role of IUS in the evaluation of IBD activity, this modality is emerging in the evaluation of treatment response and disease prognosis in pediatric IBD. Already in 2023, expert consensus suggested a monitoring algorithm using IUS in pediatric IBD [4], involving measurement at baseline, every 4–12 weeks after treatment initiation or change and every 3–6 months once the therapeutic target has been achieved. This approach is also being evaluated as a basis for national guidelines outside of Europe [53]. Studies validating this approach in clinical decision making have thus far been scarce in children. Table 4 summarizes recent evidence on this topic and is discussed below. A recently published study of the “RAINBOW” group investigated how treatment decisions would be influenced by a point-of-care ultrasound (POCUS) in 76 pediatric CD patients. The POCUS included an IUS scanning the jejunum, ileum, TI, and colon as well as determining the PCD-US score. The authors reported a preferred change in patient management in 58% of cases, with most cases requiring further diagnostic evaluation or escalating treatment. Importantly, abnormal POCUS (defined as BWT > 3.7 mm) had a high positive predictive value for clinical disease flares, especially if combined with an abnormal FCP or an abnormal mucosal inflammation non-invasive index (MINI index) [54]. A small study evaluated the predictive value of combined non-invasive methods (IUS, MRE, and video capsule endoscopy [VCE]) in 45 pediatric IBD patients in whom therapy adjustments were made based on clinical, laboratory and non-invasive diagnostic findings alone. An accuracy of 83%, a high positive predictive value of 92% but a relatively low negative predictive value of 70% were reported for achieving clinical and endoscopic remission at 52 weeks [55]. In a study involving 32 children with CD, IUS was performed at diagnosis and every three months under therapy. Notably, BWT of the TI normalized slower than the clinical disease activity index PCDAI, but quicker than FCP. Also, BWT correlated better with endoscopy (SES-CD) than PCDAI or FCP [56]. In a recently presented study of 59 children with CD, the Simple Ultrasound Activity Score for Crohn’s disease (SUS-CD [57], validated so far in adults only) was used as an endpoint to monitor transmural response or remission on biologic therapy [58]. A multicenter prospective study evaluated colonic BWT, BWS, and Doppler, as well as the MUC score as measured by IUS in a cohort of 60 pediatric patients with acute severe ulcerative colitis (ASUC) [59]. A BWT of > 5 mm measured in the lower left quadrant, and a MUC score of > 7.8 within 48 h of initiating corticosteroids were determined as optimal cut-offs for predicting steroid resistance. On days 5–7 of therapy, a BWT > 4.8 mm and a MUC > 8.7 in the left-lower quadrant were associated with therapy failure and colectomy. In acute severe ulcerative colitis (ASUC), the recently published ECCO-ESGAR-ESP-IBUS guideline lists IUS as an equal alternative to endoscopy and histology in UC, and as an equal alternative to MRE in CD to evaluate treatment response at 12 weeks after initiation of therapy [60]. The updated guidelines of ESPGHAN and ECCO on the management of pediatric UC [61] and acute severe colitis [62] now both include conditional recommendations for the use of IUS in initial evaluation and/or treatment response monitoring.

**Table 4:** Overview of recent studies addressing the role of IUS in pediatric IBD monitoring

Study [reference]	Population (*n*)	Method of IUS, score, additional modality	Comparator(s)	Key findings
RAINBOW group [26]	CD (76)	POCUS (jejunum, ileum, TI, colon); PCD-US score	Clinical assessment	IUS findings led to changed management in 58%; abnormal POCUS (BWT > 3.7 mm) had high PPV for flares, especially combined with abnormal FCP or MINI
Vela et al. [55]	IBD (45)	IUS + MRE + VCE	Clinical and laboratory findings	83% accuracy, 92% PPV, 70% NPV for clinical/endoscopic remission at 52 weeks
Grabovski et al. [56]	CD (32)	IUS every 3 months; BWT of TI	PCDAI, FCP, endoscopy (SES-CD)	TI BWT normalized slower than PCDAI but faster than FCP; BWT correlated better with SES-CD than PCDAI or FCP
deBruyn et al. [58]	CD (59)	SUS-CD score	Biologic therapy response	SUS-CD used to monitor transmural response/remission on biologics
ASUC-US Study [59]	UC (60)	BWT, BWS, Doppler and MUC Score	Patient outcome in ASUC	Within 48 h of treatment, LLQ BWT > 5 mm and MUC > 7.8 predicts steroid failure. On day 5–7 LLQ BWT > 4.8 mm and MUC > 8.7 predict colectomy

*CD*, Crohn’s disease; *IBD*, inflammatory bowel disease; *UC*, ulcerative colitis; *POCUS*, point-of-care ultrasound; *PCD-US*, pediatric Crohn’s disease ultrasound score; *IUS*, intestinal ultrasound; *MRE*, magnetic resonance enterography; *VCE*, video capsule endoscopy; *BWT*, bowel wall thickness; *TI*, terminal ileum; *SUS-CD*, simple ultrasound activity score for Crohn’s disease; *BWS*, bowel wall stratification; *MUC*, Milan ultrasound criteria; *PCDAI*, pediatric Crohn’s disease activity index; *FCP*, fecal calprotectin; *SES-CD*, simple endoscopic score for Crohn’s disease; *ASUC*, acute severe ulcerative colitis; *PPV*, positive predictive value; *MINI*, mucosal inflammation non-invasive index; *NPV*, negative predictive value; *LLQ,* *left lower quadrant*

## Discussion

Here, we provided an updated overview of recently accumulated evidence regarding the use of IUS in pediatric IBD. The studies reviewed in this work repeatedly found an IUS specificity and sensitivity for intestinal inflammation of over 80% for the bowel segments more accessible to ultrasound (colon, TI) when compared to endoscopy [8–10]. For the evaluation of the small intestine, IUS accuracy is more limited when compared to MRE and VCE based on current evidence [15, 17]. For this reason, IUS may perform better in pediatric UC and CD affecting the TI and colon than in proximal CD [10].

Recent research focused on determining cut-off values for inflamed vs. healthy bowel segments in pediatric IBD. The evidence reviewed here including a study by the pediatric committee of the International Bowel Ultrasound (IBUS) Group [32] points to a new consensus cut-off of < 2.5 mm for BWT in children, noting that this is less than the accepted < 3 mm for adults with IBD [8, 16, 17, 30–32, 32–34, 38]. For younger children including the VEO-IBD age group, the cut-off maybe even lower [40, 41].

From the currently available IUS-based scoring systems, UC-IUS was found to have a superior predictive value than the Civitelli score in two pediatric UC cohorts, and outperformed SPAUSS [30, 50]. In CD, PCD-US and BWT had the best performance [37]. An interesting finding of multiple recent studies in [30, 34, 37, 38] was the very good predictive value of BWT alone, even as compared to more complex scores [30, 37] in both CD and UC.

The quantification of mesenteric or creeping fat in pediatric IBD is an interesting emerging research topic [44]. Standardization is urgently required, and recent advances such as the development of the CMFI [45, 46] are likely to move the field forward soon. While novel sonographic parameters, such as the CMFI, may improve specificity, they also add complexity. A simplification of the ultrasound technique to include less parameters will likely make this modality more readily accessible as a bedside monitoring tool. Considering the good performance of single parameter IUS assessment of BWT alone in the literature reviewed here, future studies will have to address the “trade-off” of improved specificity in multiparameter IUS with regards to issues of accessibility, technical and training requirements and reproducibility.

IUS seems to be useful to identify bowel stenosis especially in the TI, even if it lacks specificity in differentiating inflammatory from fibrotic stenosis [48, 49], but pediatric data in this regard is lacking.

## Conclusions

Standardization and validation of IUS in the diagnostics and follow-up of pediatric IBD has made impressive recent advances, even though defining technical standards is an ongoing effort (Fig. 1). Based on available data, consensus guidelines already recommend the incorporation of ultrasound in the clinical decision-making process for pediatric IBD, however its adoption may be limited by availability and training. Structured training programs (such as the IBUS Group Training Curriculum [63]) and recent efforts to standardize and simplify IUS should lead to widespread adoption as a bedside tool in pediatric IBD medicine. Further high-quality evidence will likely emerge from prospective treatment follow-up studies including IUS-based endpoints.

## Data Availability

No datasets were generated or analysed during the current study.

## Abbreviations

AI: Artificial intelligence
ASUC: Acute severe ulcerative colitis
BWT: Bowel wall thickness
BWS: Bowel wall stratification
CD: Crohn’s disease
CDS: Color Doppler signal
CMFI: Chicago Mesenteric Fat Index
DDW: Digestive Disease Week
ECCO: European Crohn’s and Colitis Organisation
ESPGHAN: European Society of Gastroenterology, Hepatology and Nutrition
FCP: Fecal calprotectin
IBD: Inflammatory bowel disease
IBUS: International Bowel Ultrasound Group
IBUS-SAS: International Bowel Ultrasound Segmental Activity Score
IUS: Intestinal ultrasound
MRE: Magnetic resonance enterography
MINI-index: Mucosal Inflammation Non-Invasive Index
MUC: Milan Ultrasound Criteria
PCDAI: Pediatric Crohn’s Disease Activity Index
PCD-US: Pediatric Crohn’s Disease Intestinal Ultrasound Score
PIBD: Pediatric Inflammatory Bowel Disease
POCUS: Point-of-Care Ultrasound
PUCAI: Pediatric Ulcerative Colitis Activity Index
saBWT: Sum of Adjusted Bowel Wall Thickness
SICUS: Small Intestinal Contrast Ultrasonography
SMT: Submucosal thickness
SPAUSS: Simple Pediatric Activity Ultrasound Score
SUS-CD: Simple Ultrasound Activity Score for Crohn’s Disease
TI: Terminal ileum
UC: Ulcerative colitis
UC-IUS: Ulcerative Colitis Intestinal Ultrasound Score
UC-US: Ulcerative Colitis Ultrasound Score
UCEIS: Ulcerative Colitis Endoscopic Index of Severity
UEGW: United European Gastroenterology Week
VCE: Video capsule endoscopy
VEO-IBD: Very early onset inflammatory bowel disease
